# Artificial Intelligence-Assisted Quantification of Longitudinal HRCT Changes During Treatment of Pulmonary Tuberculosis: An Exploratory Proof-of-Concept Study

**DOI:** 10.3390/diagnostics16121822

**Published:** 2026-06-12

**Authors:** Anna Russo, Vittorio Patanè, Francesco Ruotolo, Maria Chiara Brunese, Mariateresa Del Canto, Loredana Alessio, Caterina Monari, Nicola Coppola, Alfonso Reginelli

**Affiliations:** 1 Precision Medicine Department, University of Campania “Luigi Vanvitelli”, 80138 Naples, Italy; 2 Radiology Department, San Leonardo Hospital, 80053 Castellamare di Stabia, Italy; 3 Dipartimento di Salute Mentale e Fisica e Medicina Preventiva, UOC Malattie Infettive, Università Degli Studi Della Campania ‘Luigi Vanvitelli’, 80138 Naples, Italy

**Keywords:** pulmonary tuberculosis, artificial intelligence, high-resolution computed tomography, computed tomography, longitudinal imaging, treatment monitoring, post-tuberculosis lung disease, radiology

## Abstract

**Background:** Treatment monitoring in pulmonary tuberculosis increasingly requires assessment of residual inflammatory burden and structural lung damage beyond microbiologic response alone. High-resolution computed tomography (HRCT) can provide this information, but interpretation of serial examinations is time-consuming and partly subjective. This study did not aim to evaluate AI for the diagnosis of pulmonary tuberculosis. Instead, it explored whether artificial intelligence (AI)-assisted quantitative HRCT analysis could support longitudinal assessment of treatment-related imaging changes in patients with microbiologically confirmed pulmonary tuberculosis. **Methods:** We conducted a retrospective, single-center, exploratory longitudinal study of patients receiving treatment for pulmonary tuberculosis. HRCT examinations acquired at diagnosis and during follow-up were anonymized, reviewed by an expert thoracic radiologist, and processed using AVIEW Lung Texture (Coreline Soft v2.0). The software quantified total lung volume and six predefined parenchymal categories: normal lung, ground-glass opacity, consolidation, reticulation, honeycombing, and emphysema. **Results:** Ninety-six patients contributed 256 HRCT examinations. The most frequent software-detected abnormalities were ground-glass opacity, consolidation, and emphysema-labeled low-attenuation areas. Ground-glass opacity and consolidation showed the clearest decline across serial examinations, consistent with regression of active inflammatory disease during treatment. Reticulation showed a heterogeneous course, likely reflecting both inflammatory resolution and residual structural remodeling. Honeycombing was infrequent and quantitatively limited. Lung volume changed variably and did not consistently parallel visual improvement. A key methodological limitation was the absence of a dedicated cavity class. As a result, emphysema-labeled low-attenuation areas should not be interpreted as conventional emphysema alone, because tuberculous cavities and post-destructive abnormalities were frequently included in this category. **Conclusions**: AI-assisted HRCT quantification may support longitudinal assessment of pulmonary tuberculosis by providing structured and reproducible measures of interval change. However, tuberculosis-specific interpretation remains dependent on expert radiologic oversight, particularly in cavitary disease.

## 1. Introduction

Tuberculosis remains one of the most consequential infectious diseases worldwide despite the availability of effective antimicrobial therapy [1,2,3]. Pulmonary tuberculosis is the most common clinical presentation and continues to account for substantial transmission, morbidity, and mortality, particularly in settings characterized by delayed diagnosis, incomplete treatment, or social vulnerability [4,5,6,7].

From an imaging standpoint, pulmonary tuberculosis is highly heterogeneous. Active disease may manifest as nodules, tree-in-bud opacities, consolidations, cavitation, or mixed interstitial and alveolar abnormalities, whereas treated or chronic disease may evolve toward fibrosis, bronchiectasis, paracicatricial emphysema, architectural distortion, and volume loss [8,9,10,11]. This morphologic diversity is clinically important but methodologically challenging, because the same patient may simultaneously show features of active inflammation and residual structural damage [12].

Although chest radiography remains central to first-line evaluation, computed tomography—and especially high-resolution computed tomography (HRCT)—provides greater sensitivity for cavitary disease, bronchogenic spread, small nodules, and subtle parenchymal or structural abnormalities. In clinical practice, HRCT is particularly useful when radiographic findings are equivocal, complications are suspected, or follow-up decisions require detailed assessment of disease extent and residual lung damage [13,14,15,16,17,18,19,20,21].

This creates an unmet clinical need in treated pulmonary tuberculosis. For many patients, the central challenge is no longer limited to detecting disease, but to understanding how disease burden changes over time: whether inflammatory abnormalities are regressing, whether cavitary destruction is stabilizing or persisting, and whether the dominant trajectory is one of healing or irreversible post-tuberculous structural damage [22,23]. These questions are highly relevant in respiratory practice because treatment monitoring increasingly extends beyond microbiologic response alone and intersects with the recognition of post-tuberculosis lung disease [24,25,26,27].

At the same time, interpretation of serial HRCT examinations is labor-intensive and partly subjective. Qualitative descriptions alone may be insufficient to capture subtle interval changes in lesion burden across multiple lobes and time points. This is relevant not only for radiologists, but also for pulmonologists involved in treatment monitoring, because structured and reproducible summaries of interval change may facilitate multidisciplinary assessment and longitudinal decision-making [28,29,30,31,32].

In recent years, artificial intelligence (AI) has been increasingly applied to thoracic imaging. In tuberculosis, most AI studies have focused on chest radiography for screening, triage, or diagnostic support. However, longitudinal treatment monitoring represents a different clinical task: rather than determining whether tuberculosis is present, it requires assessment of interval change, inflammatory regression, cavitary evolution, and residual structural remodeling. This CT-based follow-up application remains comparatively underexplored [33,34,35,36]. The latter remains a comparatively underexplored area.

The rationale for quantitative CT support is strengthened by experience from other diffuse lung diseases, where AI-assisted HRCT quantification has been proposed to improve standardization, support longitudinal monitoring, and complement visual interpretation [37,38]. Additional support for the clinical relevance of AI-derived CT quantification comes from infectious lung disease outside tuberculosis. In hospitalized patients with COVID-19, Parczewski et al. reported that artificial neural network-derived percentage of lung tissue involvement on CT was independently associated with in-hospital mortality and risk of mechanical ventilation [36]. Although COVID-19 and tuberculosis differ substantially in pathophysiology, time course, and imaging patterns, this study supports the broader concept that quantitative CT-derived measures of lung involvement may provide clinically meaningful information when integrated with expert interpretation and clinical data [36]. Nevertheless, pulmonary tuberculosis presents additional challenges because it combines inflammatory, nodular, cavitary, fibrotic, and airway-centered abnormalities within the same patient and often within the same region of lung [39,40,41]. For this reason, any application of generic texture-analysis software to tuberculosis must be judged not only by its ability to assign labels, but also by the clinical interpretability of those labels within a disease-specific morphologic context.

The present study was motivated by a practical clinical question. Rather than developing a tuberculosis-specific algorithm, we explored the use of a commercially available texture-analysis platform, AVIEW Lung Texture (Coreline Soft), in a cohort of patients with pulmonary tuberculosis undergoing serial HRCT during therapy. The aim was not to claim autonomous diagnosis, nor to report de novo algorithm development, but to evaluate whether structured AI-derived quantification could meaningfully complement expert radiologist assessment during longitudinal follow-up and provide clinically useful information for respiratory physicians monitoring treatment response.

Accordingly, the purpose of this study was to describe the longitudinal HRCT patterns generated by AVIEW Lung Texture in treated pulmonary tuberculosis and to assess their interpretive value in relation to disease regression, residual remodeling, and lobar distribution, while also identifying the principal strengths and limitations of repurposing a non-tuberculosis-specific texture analysis platform for this clinical indication. The primary exploratory endpoint was the longitudinal change in AI-derived whole-lung and lobar percentages of ground-glass opacity, consolidation, reticulation, honeycombing, emphysema-labeled low-attenuation areas, normal parenchyma, and total lung volume between baseline and follow-up HRCT examinations. Thus, the focus of the study was treatment monitoring and characterization of longitudinal HRCT changes in confirmed pulmonary tuberculosis, rather than AI-based diagnosis or screening.

## 2. Materials and Methods

### 2.1. Study Design and Ethics

This retrospective, single-center, longitudinal observational study was conducted at the University Hospital “Luigi Vanvitelli”, in Naples, Italy. The study was approved by the Ethics Review Committee “L. Vanvitelli” and AORN “Ospedale dei Colli”, Naples (Protocol No. 13953/i/2022). Owing to the retrospective design and the use of anonymized imaging data, the requirement for study-specific informed consent was waived by the ethics committee. The study was based on retrospective analysis of serial HRCT examinations performed in patients receiving treatment for pulmonary tuberculosis.

### 2.2. Patient Population

Ninety-six patients with pulmonary tuberculosis were included. All included patients had microbiologically confirmed active pulmonary tuberculosis. No multidrug-resistant tuberculosis cases were included. Clinically relevant concomitant pulmonary superinfections were excluded on the basis of available clinical, microbiological, and radiologic data. The cohort comprised 64 men and 32 women, with a mean age of 40.1 years (range, 15–63 years). All patients had undergone HRCT at the time of diagnosis and at least one additional HRCT examination during follow-up while receiving anti-tuberculous therapy. HIV status and significant comorbidities were not systematically collected or analyzed, as the study was designed as an exploratory radiologic and functional imaging experience rather than as a clinical-prognostic investigation.

Forty patients contributed two CT examinations, 48 patients contributed three CT examinations, and 8 patients contributed four CT examinations, for a total of 256 HRCT studies available for analysis.

### 2.3. Image Acquisition

HRCT scans were acquired on contemporary multidetector CT systems, including a GE Revolution HD scanner (GE HealthCare, Milan, Italy) and a Siemens SOMATOM scanner (Siemens Healthineers, Erlangen, Germany), using a standardized thin-section inspiratory HRCT protocol consistent with international practice. Patients were scanned in the supine position during full-inspiration breath-hold. Volumetric thin-section datasets were reconstructed with contiguous or near-contiguous sections, without intentional interslice gaps, using a slice thickness of 1–2 mm and high-spatial-frequency lung reconstruction algorithms. Scanner-specific iterative reconstruction methods were applied according to institutional protocol. Images were reconstructed with lung window settings and archived in DICOM format within the institutional PACS. Before AI-based processing, all examinations were reviewed for adequate inspiratory effort, absence of major motion artifacts, and suitability for quantitative lung texture analysis. Examinations were performed primarily without intravenous contrast material, although contrast-enhanced scans were obtained when clinically indicated. The interval between serial CT examinations varied according to clinical evolution and treatment duration. The shortest interval was 3 months, and the average interval between studies was approximately 4–6 months, in keeping with routine timing of induction and continuation phases of anti-tuberculous therapy.

### 2.4. Radiologic Workflow and AI Software Analysis

Each HRCT examination was first reviewed and reported in routine clinical practice by a dedicated thoracic radiologist with more than 15 years of experience. The radiologic assessment reflected a real-world longitudinal follow-up workflow; therefore, the radiologist was not blinded to prior imaging examinations when evaluating interval changes. This approach was chosen because comparison with previous CT studies represents standard practice in treatment monitoring of pulmonary tuberculosis. Only after expert radiologic interpretation were the anonymized DICOM datasets processed using AVIEW Lung Texture, a module of the Coreline AVIEW platform developed by Coreline Soft. Interobserver variability was not assessed, as the study was conceived as an exploratory proof-of-concept analysis rather than as a reproducibility study of visual radiologic scoring. Accordingly, the study was not designed to measure formal AI classification accuracy against a lesion-level ground truth. No expert segmentation, multi-rater annotation, or external reference standard was available; therefore, performance metrics such as sensitivity, specificity, Dice similarity coefficient, or interobserver agreement were not calculated. The evaluation focused instead on feasibility, longitudinal consistency, and expert radiologic verification of the anatomic plausibility of software-derived labels.

For each examination, the software generated quantitative outputs for total lung volume and for the relative distribution of six predefined parenchymal categories: normal lung, ground-glass opacity, consolidation, reticulation, honeycombing, and emphysema. No additional tuberculosis-specific category was introduced because the study evaluated a commercially available software platform with a predefined texture ontology. In particular, the software did not include a dedicated cavity class. Creating such a category would have required lesion-level cavity annotation, algorithm retraining, and independent validation, which were beyond the scope of this exploratory proof-of-concept study. Outputs were expressed both as percentages of total lung volume and as lobar distributions. The platform also provided color-coded overlays on axial and coronal images, enabling direct visual comparison between software labels and the underlying CT anatomy.

This visual verification layer was considered essential in the present study because it allowed recognition of cases in which software labels did not correspond directly to tuberculosis-specific morphologic patterns.

**Figure 1:**
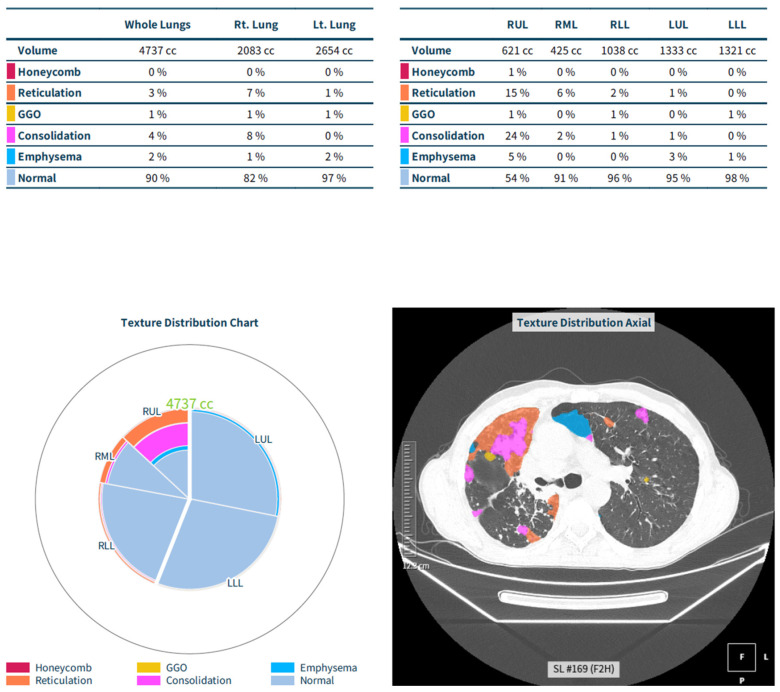
Representative AI-assisted HRCT texture analysis output in pulmonary tuberculosis. The **upper** tables show quantitative whole-lung, right/left lung, and lobar volumes, together with the percentage distribution of software-defined parenchymal texture categories. The **lower left** panel shows the texture distribution chart, while the **lower right** panel shows the corresponding axial HRCT image with color-coded texture overlay. This example illustrates how AI-derived outputs can provide a structured summary of regional disease burden, while requiring radiologist verification of software-assigned labels in tuberculosis-related abnormalities. Abbreviations: AI, artificial intelligence; HRCT, high-resolution computed tomography; Rt., right; Lt., left; RUL, right upper lobe; RML, right middle lobe; RLL, right lower lobe; LUL, left upper lobe; LLL, left lower lobe; GGO, ground-glass opacity; cc, cubic centimeters.

Representative examples of the software workflow are shown in Figure 1. The platform provided both numerical outputs, including whole-lung and lobar texture percentages, and color-coded overlays that allowed direct comparison between AI-assigned categories and the underlying HRCT morphology.

This visual verification step was essential because tuberculosis-related abnormalities may have overlapping morphologic features, and software-derived labels may not always correspond directly to disease-specific patterns such as cavitation or post-destructive low-attenuation change.

### 2.5. Outcome Measures

The main outcome measures were longitudinal changes in software-derived whole-lung and lobar percentages of ground-glass opacity, consolidation, reticulation, honeycombing, emphysema-labeled low-attenuation areas, normal parenchyma, and total lung volume. Particular attention was given to whether these changes paralleled expected treatment response or chronic structural remodeling.

### 2.6. Image Interpretation Framework

Image interpretation integrated three complementary levels of information: first, the original radiology report, which established the clinical imaging context for each examination; second, the software-generated quantitative outputs, which provided structured longitudinal data; and third, the color-coded overlays, which allowed direct verification of the anatomic correlates of each labeled pattern. This integrated approach was necessary because numerical percentages alone can be misleading in tuberculosis, where the meaning of a pattern depends heavily on lesion morphology, location, and treatment stage.

### 2.7. Statistical Analysis

The analysis was descriptive and exploratory. Continuous variables were summarized using means, ranges, and percentages, together with graphical longitudinal trajectories. Because the study was not designed or powered for inferential longitudinal modeling, formal normality testing and hypothesis testing were not performed. Confidence intervals were not calculated, and the results were interpreted as hypothesis-generating rather than confirmatory.

Longitudinal trends were evaluated by comparing sequential HRCT examinations within each patient and by summarizing software-derived whole-lung and lobar percentages across available time points. Because follow-up was heterogeneous and reflected routine clinical practice, patients were analyzed according to the available examination order, namely baseline, second, third, and fourth HRCT when present, rather than according to fixed protocol-defined time intervals. Missing follow-up examinations were not imputed; an available-case approach was used for each longitudinal comparison.

## 3. Results

### 3.1. Cohort Structure

**Figure 2:**
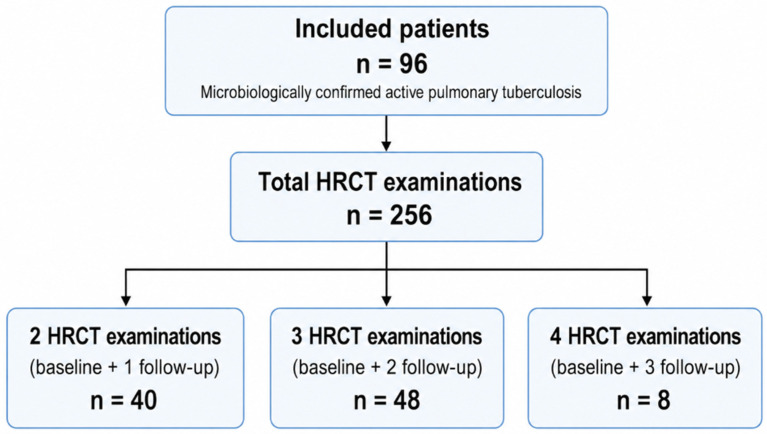
Flowchart summarizing patient inclusion and longitudinal HRCT follow-up distribution. Ninety-six patients with microbiologically confirmed active pulmonary tuberculosis were included, contributing a total of 256 HRCT examinations. Forty patients underwent two HRCT examinations, 48 underwent three HRCT examinations, and 8 underwent four HRCT examinations. Abbreviation: HRCT, high-resolution computed tomography.

The final study population included 96 patients with pulmonary tuberculosis and 256 HRCT examinations. The cohort was predominantly male (64/96, 66.7%), and the mean age was 40.1 years. g schedule: 40 patients contributed a baseline and one follow-up examination, 48 contributed a baseline and two follow-up examinations, and 8 contributed a baseline and three follow-up examinations. This cohort structure, including patient inclusion, total number of HRCT examinations, and distribution of longitudinal follow-up examinations, is summarized in Figure 2.

### 3.2. Overall Software-Derived Abnormalities

Across the cohort, the software most frequently identified ground-glass opacity, consolidation, and emphysema-labeled low-attenuation areas. Reticulation was also common, whereas honeycombing was less frequent and quantitatively limited.

Ground-glass opacity and consolidation were consistent with active inflammatory and exudative disease. By contrast, reticulation and honeycombing were more consistent with chronic remodeling and fibrotic change. The emphysema category required specific reinterpretation because the software did not distinguish tuberculous cavities from other low-attenuation lesions. As a result, the high prevalence of emphysema-labeled regions should not be interpreted as reflecting conventional emphysema alone, but rather a composite of cavitary lesions, post-destructive change, paracicatricial air-space enlargement, and true low-attenuation remodeling.

### 3.3. Longitudinal Lung Volume Change

**Figure 3:**
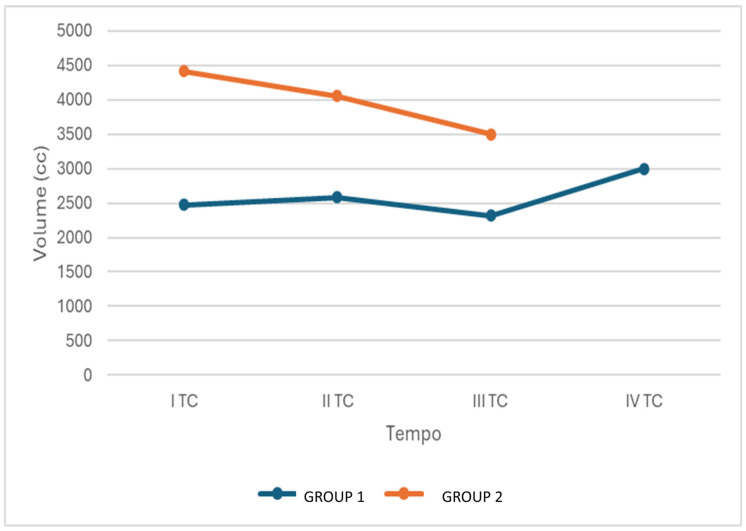
Longitudinal trends in total lung volume across serial HRCT examinations. Volumetric trajectories were variable, highlighting the complexity of interpreting lung volume in treated pulmonary tuberculosis. Abbreviation: HRCT, high-resolution computed tomography.

Total lung volume showed heterogeneous longitudinal behavior. Overall, lung volume increased across serial studies in 60% of patients and decreased in 40% (Figure 3). Because the study was based on retrospectively available serial examinations with heterogeneous follow-up timing, lung volume changes were interpreted descriptively according to the direction of change rather than through formal variability-based longitudinal modeling. Volumetric change did not consistently parallel visual reduction in disease burden.

In several patients, total or lobar lung volume decreased despite contemporaneous reduction in inflammatory lesions and increase in the proportion of normal parenchyma. This pattern is compatible with residual contraction related to fibrosis, scarring, or lobar volume loss during healing. Conversely, in other patients, increasing volume likely reflected improved aeration and re-expansion of previously affected segments. These observations indicate that lung volume, although clinically relevant, is not a sufficiently specific standalone marker of treatment response in pulmonary tuberculosis.

### 3.4. Honeycombing

Honeycombing was limited at baseline and remained quantitatively modest throughout follow-up. It was present in 20% of patients at diagnosis, with an average extent generally around 1–2% and peak values of approximately 5% in the most involved cases. Longitudinally, honeycombing increased in 20% of patients, decreased in 20%, and remained substantially stable in 60%.

**Figure 4:**
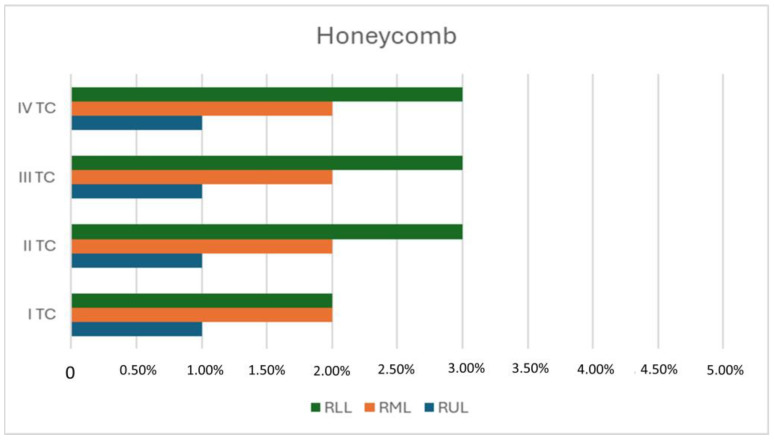
Longitudinal lobar distribution of honeycombing. The quantitative burden remained low across serial examinations, consistent with limited residual fibrotic remodeling rather than active disease.

The right upper, right middle, and right lower lobes were the most frequently involved sites (Figure 4). Even where present, the burden was limited, suggesting that the software identified small cystic fibrotic components rather than widespread end-stage fibrosing destruction.

### 3.5. Reticulation

Reticulation was more frequent than honeycombing and showed a heterogeneous temporal course. It was present in 75% of patients. Over follow-up, reticulation increased in 40% of cases, decreased in 40%, and remained stable in 20%.

**Figure 5:**
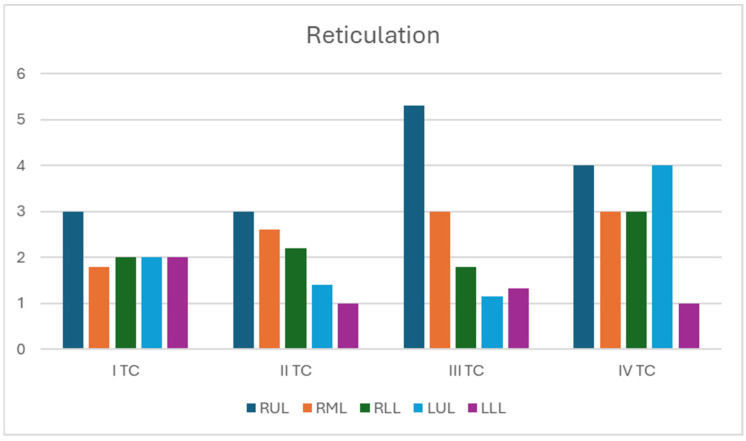
Longitudinal lobar distribution of reticulation. Reticular abnormalities showed a heterogeneous temporal course, with upper-lobe predominance and variable interval behavior across follow-up.

The right upper lobe was the most frequently involved site, with values reaching approximately 15% in some patients, whereas the left lower lobe generally showed lower percentages, often in the range of 1–2% (Figure 5). This variable behavior suggests that reticulation in treated tuberculosis may represent a mixed imaging pattern, potentially related to resolving inflammatory interstitial change, residual architectural distortion, or evolving structural remodeling. In the absence of histopathologic correlation, however, reticulation cannot be definitively classified as established fibrosis.

### 3.6. Ground-Glass Opacity

Ground-glass opacity was one of the most prominent and dynamically informative abnormalities identified by the software. It was frequently observed in the lower lobes, particularly in the left lower lobe and right lower lobe, where mean values progressively declined over serial examinations.

**Figure 6:**
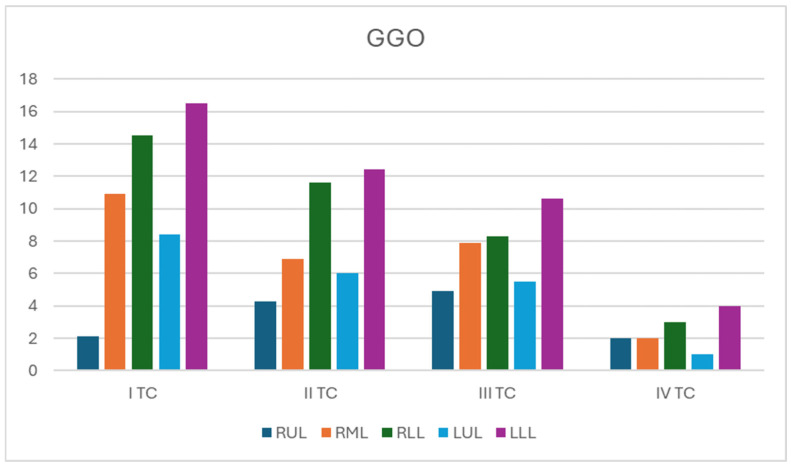
Longitudinal lobar distribution of ground-glass opacity. Mean percentages progressively declined across serial HRCT examinations, particularly in the lower lobes, supporting its value as a marker of decreasing inflammatory burden. Abbreviations: GGO, ground-glass opacity; HRCT, high-resolution computed tomography.

Mean ground-glass opacity in the left lower lobe decreased from 16.5% at baseline to 12.4%, 10.6%, and 4.0% on subsequent examinations. Corresponding values in the right lower lobe decreased from 14.5% to 11.6%, 8.3%, and 3.0% (Figure 6). Among the available software outputs, ground-glass opacity showed one of the clearest longitudinal patterns of regression and therefore appeared to be a useful quantitative correlate of decreasing inflammatory burden during treatment. This finding represents one of the most robust longitudinal signals observed in the cohort, because the reduction in ground-glass opacity was directionally consistent across serial examinations and anatomically coherent with visual improvement in inflammatory parenchymal abnormalities.

### 3.7. Consolidation

**Figure 7:**
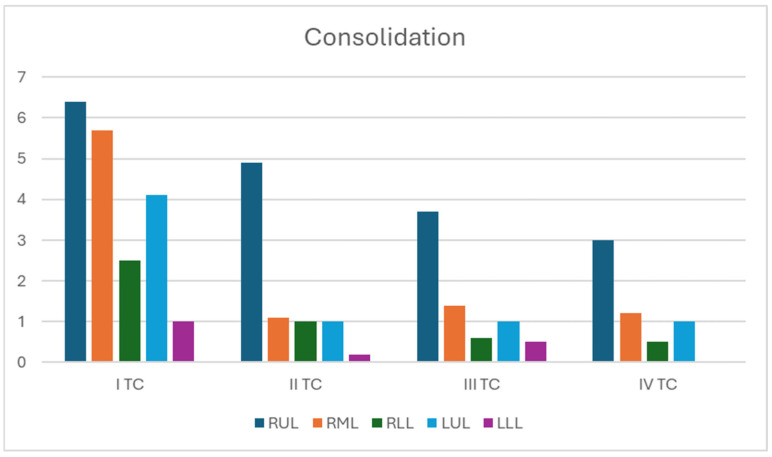
Longitudinal lobar distribution of consolidation. Consolidative burden decreased across serial HRCT examinations, most prominently in initially involved right upper and right middle lobes. Abbreviation: HRCT, high-resolution computed tomography.

Consolidation was also a major abnormality category at baseline and generally regressed during treatment. The most involved lobes were the right upper lobe and right middle lobe, with peak values reaching approximately 24% and 40%, respectively. Across serial examinations, consolidative burden typically declined and, in some patients, disappeared completely (Figure 7).

Compared with other software-defined patterns, consolidation was among the most straightforward to interpret clinically. Its reduction over time broadly paralleled expected resolution of active alveolar inflammatory involvement under anti-tuberculous therapy.

### 3.8. Emphysema-Labeled Low-Attenuation Areas

**Figure 8:**
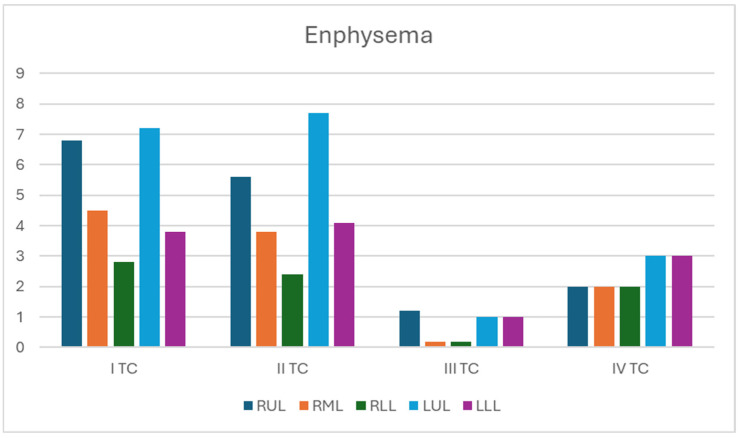
Longitudinal lobar distribution of emphysema-labeled low-attenuation areas. The irregular temporal behavior of this category reflects the software’s grouping of heterogeneous destructive lesions, including cavitary disease, rather than conventional emphysema alone.

The emphysema output was among the most abundant but also the most difficult to interpret categories. Emphysema-labeled low-attenuation areas showed complex temporal behavior. On the second CT examination, values decreased in the right upper, middle, and lower lobes but increased simultaneously in the left lobes. On the third examination, the software reported a marked decrease across all lobes, followed in some cases by a slight increase at the fourth follow-up study (Figure 8).

These fluctuations were not consistent with the expected behavior of true progressive emphysema. Instead, visual review of the overlays and source images indicated that the software frequently grouped tuberculous cavities and other destructive low-attenuation lesions within the emphysema category. Accordingly, this output should be interpreted as a generic low-attenuation abnormality rather than as a direct surrogate for conventional emphysema. This represents one of the main interpretive limitations of applying a non-tuberculosis-specific texture-analysis platform to longitudinal tuberculosis imaging.

### 3.9. Integrated Longitudinal Interpretation

Taken together, the serial trends observed in the cohort supported a coherent longitudinal pattern. Ground-glass opacity and consolidation tended to decline during treatment, consistent with regression of active inflammatory disease. Reticulation showed mixed behavior, likely reflecting the coexistence of healing and residual scarring. Honeycombing was infrequent and mainly reflected established structural damage, whereas lung volume changed variably according to the balance between improved aeration and residual contraction. Emphysema-labeled low-attenuation areas captured clinically relevant destructive abnormalities but were semantically confounded by cavitary disease.

**Figure 9:**
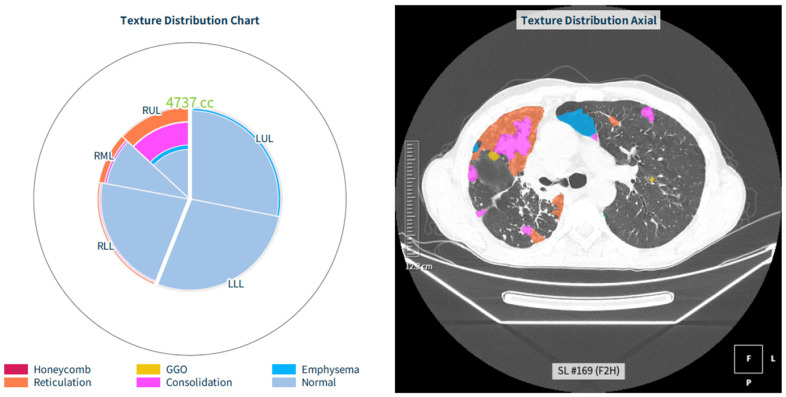
Representative AI-assisted HRCT texture analysis during pulmonary tuberculosis follow-up. The **left** panel shows the texture distribution chart, while the **right** panel shows the corresponding axial HRCT image with color-coded texture overlay. This example illustrates the use of AI-derived quantitative outputs to summarize regional disease burden during follow-up, while emphasizing the need for radiologist verification of software-assigned labels in cavitary and destructive tuberculosis-related abnormalities. Abbreviations: AI, artificial intelligence; HRCT, high-resolution computed tomography; Rt., right; Lt., left; RUL, right upper lobe; RML, right middle lobe; RLL, right lower lobe; LUL, left upper lobe; LLL, left lower lobe; GGO, ground-glass opacity; cc, cubic centimeters.

**Figure 10:**
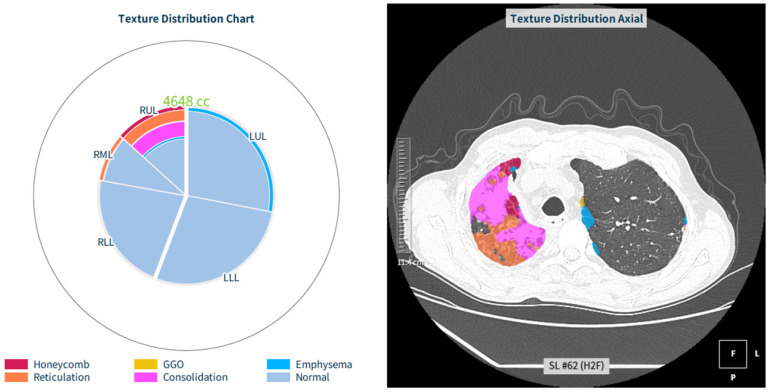
AI-assisted HRCT texture analysis showing regional parenchymal involvement during pulmonary tuberculosis follow-up. The left panel summarizes texture distribution by lobe, and the lower right panel shows the corresponding axial HRCT image with color-coded overlay. The figure highlights the ability of AI-derived outputs to localize residual abnormalities, particularly in the right upper lobe, while reinforcing the need for radiologist verification of software labels in tuberculosis-related cavitary or destructive lesions. Abbreviations: AI, artificial intelligence; HRCT, high-resolution computed tomography; Rt., right; Lt., left; RUL, right upper lobe; RML, right middle lobe; RLL, right lower lobe; LUL, left upper lobe; LLL, left lower lobe; GGO, ground-glass opacity; cc, cubic centimeters.

Representative longitudinal examples are shown in Figure 9 and Figure 10. These examples illustrate how AI-assisted HRCT texture analysis can summarize interval changes in regional disease burden, lung volume, and software-defined parenchymal categories across treatment follow-up. The color-coded overlays were used to verify the morphologic meaning of software-derived labels, particularly in cavitary and destructive tuberculosis-related abnormalities.

## 4. Discussion

This exploratory study evaluated a commercial AI-based CT texture analysis platform for longitudinal HRCT follow-up in patients receiving treatment for pulmonary tuberculosis. The main finding is that AI-derived quantification may provide a structured adjunct to radiologist interpretation, particularly for tracking temporal changes in ground-glass opacity, consolidation, and overall lesion burden. From a clinical respiratory perspective, these outputs may help pulmonologists compare serial examinations more consistently and support multidisciplinary discussion during follow-up. Quantitative assessment may be clinically helpful because it transforms serial HRCT comparison from a purely qualitative evaluation into a structured summary of interval change. In particular, decreasing ground-glass opacity and consolidation may support radiologic impression of inflammatory regression, whereas reticulation and lung volume changes may help describe residual remodeling, improved aeration, or volume loss. These metrics should not be interpreted as independent response criteria, but as adjunctive information that can improve consistency of radiology reporting and facilitate communication with pulmonologists during treatment follow-up However, the clinical meaning of software-defined categories in tuberculosis remains dependent on expert morphologic interpretation.

The most clinically informative outputs were ground-glass opacity and consolidation. Both patterns showed progressive decline during follow-up and broadly paralleled expected radiologic improvement under effective anti-tuberculous therapy. Among these two patterns, ground-glass opacity was particularly informative because it showed a clear longitudinal reduction, especially in the lower lobes, supporting its role as a sensitive quantitative marker of decreasing inflammatory burden during treatment follow-up. This is precisely the context in which quantitative serial imaging may offer added value. In routine practice, comparison across multiple prior CT studies can be cognitively demanding, especially when abnormalities are bilateral, multifocal, and partially overlapping. A platform that summarizes lesion burden by category and by lobe may therefore improve consistency in longitudinal assessment and facilitate communication between radiologists and respiratory physicians.

These findings are consistent with the broader AI literature in tuberculosis, although most previous studies have focused on chest radiography for screening, triage, or diagnostic support. By contrast, longitudinal CT-based monitoring during treatment remains less developed. Our results suggest that AI may also have value beyond disease detection, by helping clinicians characterize interval change during follow-up [42,43,44,45,46,47].

This potential value is clinically relevant because respiratory follow-up in pulmonary tuberculosis increasingly extends beyond microbiologic response alone. Post-tuberculosis lung disease may include fibrosis, bronchiectasis, emphysematous destruction, architectural distortion, and chronic volume loss. In this setting, serial HRCT can help distinguish inflammatory regression from persistent structural injury.

Experience from other diffuse lung diseases supports the use of quantitative HRCT to standardize longitudinal assessment. However, tuberculosis is more morphologically heterogeneous; therefore, AI-derived outputs must be interpreted within a disease-specific radiologic framework.

Reticulation and honeycombing provided imaging clues to residual structural damage, but their interpretation requires caution. Pulmonary tuberculosis may evolve with fibrosis, bronchiectasis, architectural distortion, air-space enlargement, and regional volume loss; however, CT reticulation alone does not allow a definitive distinction between resolving inflammatory interstitial abnormality and established fibrotic remodeling. In this cohort, the heterogeneous behavior of reticulation was therefore interpreted as a non-specific longitudinal imaging pattern rather than as a direct histopathologic marker of fibrosis. Honeycombing was infrequent and limited, consistent with its role as a marker of established fibrotic remodeling rather than active infection. Total lung volume was also complex: increasing volume may reflect improving aeration, whereas decreasing volume may occur despite treatment response because of permanent contraction, scarring, or lobar collapse. Volumetric analysis is therefore informative only when interpreted together with lesion-specific changes and direct image review.

The most important limitation was misclassification of tuberculous cavities. Cavitation is one of the most clinically relevant imaging features of post-primary tuberculosis, with implications for bacillary burden, transmissibility, and structural destruction. However, the software lacked a dedicated cavity category and frequently labeled cavitary lesions as emphysema. This limitation reflects the use of a non-tuberculosis-specific commercial texture-analysis platform rather than a tuberculosis-tailored algorithm. Therefore, the emphysema output should be regarded as a low-attenuation/destructive abnormality flag requiring expert radiologic interpretation, not as a specific diagnosis of conventional emphysema. This is not merely semantic: if interpreted naively, the output could be clinically misleading. In this cohort, the emphysema label functioned better as a flag for low-attenuation abnormality than as a disease-specific diagnosis.

This issue also has methodological implications. Prior CT-based tuberculosis studies have shown that automated or semi-automated analysis can derive disease-burden biomarkers and that cavity-specific quantification is feasible when the ontology is explicitly designed around tuberculosis morphology. Our results reach a similar conclusion from the opposite angle: a generic texture-analysis platform may capture broad longitudinal trends, but when disease-specific categories such as cavitation are absent, the limits of semantic transfer become evident. Clinically useful AI in tuberculosis must therefore be judged not only by whether it produces numbers, but by whether those numbers preserve the morphologic meaning of the lesions measured.

For this reason, the present findings do not support autonomous interpretation by non-radiologists. Rather, they support a supervised model in which AI-derived summaries make serial imaging information more structured, reproducible, and accessible to pulmonologists, while expert radiologic oversight remains essential whenever morphology has diagnostic or management implications. This is particularly important in tuberculosis, where cavitary disease, nodular bronchogenic spread, and mixed chronic sequelae cannot be reduced to generic percentages alone.

The study has several limitations. First, its retrospective single-center design may have introduced selection bias, and the relatively small cohort limits generalizability. Second, no external validation cohort was available, and there was no lesion-level reference standard, such as expert segmentation or consensus multi-reader annotation. Consequently, the actual classification accuracy of the software could not be formally quantified, and no performance metrics such as sensitivity, specificity, Dice similarity coefficient, or interobserver agreement could be calculated. Third, follow-up HRCT examinations were performed according to clinical need rather than at protocol-defined time points, resulting in heterogeneous acquisition timing across patients. Fourth, although all patients received anti-tuberculous therapy, potential treatment heterogeneity could not be fully accounted for in this imaging-focused analysis. Finally, the software ontology was not tuberculosis-specific and lacked dedicated categories for cavities, nodules, or tree-in-bud abnormalities, limiting pattern assignment in a disease characterized by mixed morphologies. Despite these limitations, the study provides clinically relevant insights. AI-generated quantification appeared most useful for tracking broad inflammatory patterns such as ground-glass opacity and consolidation. Structured whole-lung and lobar outputs may also help identify evolving fibrotic remodeling that is difficult to summarize qualitatively. At the same time, tuberculosis remains a morphology-dependent disease in which software percentages cannot be interpreted outside anatomic context.

Future work should focus on multicenter validation across scanner platforms and epidemiologic settings, refinement of the imaging ontology to include cavities and tuberculosis-specific nodular or bronchogenic spread patterns, multimodal correlation with microbiologic, functional, and prognostic outcomes, and workflow integration with explainable visual outputs that remain transparent to radiologists and useful to pulmonologists.

Overall, these findings support a balanced interpretation. AI is neither a replacement for radiologic judgment nor a negligible add-on. In pulmonary tuberculosis follow-up, its current value lies in providing a structured and reproducible quantitative layer over complex serial HRCT data, provided that all outputs are interpreted within expert morphologic context.

## 5. Conclusions

In this exploratory single-center longitudinal study, AI-assisted CT texture analysis provided standardized quantitative information that may support radiologist assessment of pulmonary tuberculosis during treatment follow-up. Serial changes in ground-glass opacity and consolidation were the most informative markers, generally reflecting regression of active inflammatory disease, while reticulation, honeycombing, and lung volume changes captured more complex aspects of residual structural remodeling.

These findings suggest that AI-derived HRCT metrics may help make longitudinal imaging assessment more reproducible and accessible within multidisciplinary respiratory care. However, tuberculosis-specific interpretation remains essential. The most important limitation was the lack of a dedicated cavity class, which led to frequent classification of tuberculous cavities and post-destructive low-attenuation abnormalities as emphysema-labeled areas. This finding highlights a major limitation of non-tuberculosis-specific AI software: quantitative outputs may capture relevant destructive abnormalities, but their semantic meaning may be misleading unless verified by expert radiologic assessment. Overall, AI-assisted texture analysis should be viewed not as an independent diagnostic or staging tool, but as a promising adjunct to human interpretation. Larger multicenter studies are needed to validate tuberculosis-specific reference standards and to correlate quantitative imaging metrics with microbiologic, functional, and prognostic outcomes. Such studies should include expert lesion-level annotation and multi-reader assessment to determine the true accuracy and reproducibility of AI-derived tuberculosis imaging classifications.

## Data Availability

Data available on demand to the corresponding author.

## Abbreviations

The following abbreviations are used in this manuscript:

AI	artificial intelligence
AORN	Azienda Ospedaliera di Rilievo Nazionale
cc	cubic centimeters
CT	computed tomography
DICOM	Digital Imaging and Communications in Medicine
GE	General Electric
GGO	ground-glass opacity
HD	high definition
HRCT	high-resolution computed tomography
LLL	left lower lobe
Lt.	left
LUL	left upper lobe
PACS	Picture Archiving and Communication System
RLL	right lower lobe
RML	right middle lobe
Rt.	right
RUL	right upper lobe
